# Antimicrobial Evaluation and Fraction-Based Profiling of Basil Essential Oil Against Vaginal Pathogens

**DOI:** 10.3390/antiox14060628

**Published:** 2025-05-23

**Authors:** Minkyoung Park, Jumin Park, Dae Youn Hwang, Sohae Park, Heeseob Lee

**Affiliations:** 1 Department of Food Science and Nutrition, College of Human Ecology, Pusan National University, Busan 46241, Republic of Korea; robina56@pusan.ac.kr (M.P.); juminpark@pusan.ac.kr (J.P.); 2 Department of Biomaterials Science, College of Natural Resources and Life Science, Pusan National University, Miryang 50463, Republic of Korea; dyhwang@pusan.ac.kr; 3 Longevity & Wellbeing Research Center, Pusan National University, Miryang 50463, Republic of Korea

**Keywords:** natural therapeutic agent, phytochemical analysis, antimicrobial effects, bioactive compounds, ethnopharmacology, traditional medicine

## Abstract

Basil (*Ocimum basilicum* L.) has been traditionally used in various cultures for its medicinal properties. This study evaluated the antioxidant and antimicrobial activities of basil essential oil (BEO) and identified its key bioactive compounds. Antioxidant activity testing, as determined by DPPH and ABTS assays, returned EC_50_ values of 115.36 and 54.77 µg/mL, respectively. BEO demonstrated significant antimicrobial effects against *Gardnerella vaginalis*, *Fannyhessea vaginae*, *Chryseobacterium gleum*, and *Candida albicans*, with inhibition zones of up to 25.88 mm and MIC values ranging from 31 to 500 µg/mL. GC-MS (gas chromatography–mass spectrometry) identified monoterpene, phenylpropene, and sesquiterpene derivatives in BEO. In addition, Fraction 3 (Fr. 3) obtained by preparative HPLC had the highest antimicrobial activity, and methyl trans-cinnamate was identified as the primary active compound in this fraction. BEO had no toxic effect on *Lactobacillus crispatus* or human dermal fibroblasts. These findings support the traditional use of basil and highlight its potential as a safe, natural therapeutic agent with antioxidant and antimicrobial properties.

## 1. Introduction

Traditionally utilized medicinal herbs consist of various plant species and contribute significantly to the synthesis and development of modern pharmaceuticals [1]. Particularly, in some developing countries, herbal medicines are utilized as the primary treatment for infections, and they are regarded as a valuable resource in ongoing research aimed at discovering natural antibacterial agents [2].

Basil (*Ocimum basilicum* L.), which is also one of these medicinal plants and a member of the Lamiaceae family, has been traditionally used in the folk medicines of various cultures as a tonic, anthelmintic, diuretic, and antispasmodic, and as a treatment for upper respiratory tract infections [3,4]. Furthermore, modern pharmacological studies have shown that basil has significant antibacterial, anti-inflammatory, and antioxidant activities [5,6,7]. These therapeutic properties are attributed primarily to its rich composition of bioactive compounds, which include monoterpenes, sesquiterpenes, phenylpropanoids, anthocyanins, and phenolic acids [8]. Particularly, the bioactive compounds in basil may promote antimicrobial activity by disrupting the permeability and integrity of bacterial membranes, producing intracellular ATP and potassium ion leakage, ultimately leading to cell death [9]. Thus, basil has been utilized in various ways across different regions and cultures, and its traditional uses are gradually being validated through contemporary scientific research.

Among the extracts derived from basil, basil essential oil (BEO) also has potent antimicrobial effects, particularly against Gram-positive bacteria such as *Bacillus sacharolyticus*, *B. stearothermophilus*, *B. subtilis*, *B. thuringiensis*, *Micrococcus glutamicus*, and *Sarcina lutea* [10]. Given these broad antimicrobial properties, BEO has therapeutic potential for the treatment of a variety of infections, including those affecting the vaginal microbiota. However, few studies have investigated its effects on vaginal infections, such as bacterial vaginosis and candidiasis. Thus, although the therapeutic properties of basil are well documented, the specific mechanisms underlying its antimicrobial activity, particularly in the context of vaginal health, remain insufficiently explored.

A healthy vaginal pH typically ranges from 3.8 to 4.5 and is maintained by beneficial lactobacilli such as *Lactobacillus crispatus* (*L. crispatus*), *L. jensenii*, *L. gasseri*, and *L. coleohominis* [11,12,13]. Disruptions to this acidic environment or reductions in *lactobacillus* populations can lead to vaginal dysbiosis and facilitate the growth of pathogenic microorganisms associated with bacterial vaginosis (BV) and vulvovaginal candidiasis (VVC) [14]. BV is one of the most prevalent vaginal infections in women and arises from disturbances in the vaginal ecosystem due to increases in the relative populations of bacteria such as *Gardnerella vaginalis* (*G. vaginalis*), *Atopobium vaginae* (*Fannyhessea vaginae*; *F. vaginae*), and *Chryseobacterium gleum* (*C. gleum*) [15,16]. The resulting conditions are characterized by malodorous vaginal discharge, itching, and discomfort [17]. Similarly, VVC is a common condition caused by *Candida* species, particularly *Candida albicans* (*C. albicans*), and has a lifetime prevalence of up to 75% [18].

Various therapeutic approaches, such as antimicrobial agents, probiotics, and lifestyle modifications, have been proposed to manage and prevent BV and VVC. However, the overuse of antibiotics has led to the development of antibiotic-resistant strains and the disruption of the normal vaginal microbiota, particularly to the depletion of beneficial lactic acid bacteria. These challenges highlight the need for phytotherapeutic agents with scientifically validated efficacy and safety profiles to restore vaginal health and prevent recurrent vaginitis.

This study aimed to evaluate the antioxidant and antimicrobial activities of BEO against four vaginitis-associated pathogens, viz., *G. vaginalis*, *F. vaginae*, *C. gleum*, and *C. albicans.* In addition, we identified the key active antimicrobial compounds within BEO and assessed the cytotoxicity of BEO on beneficial vaginal microbiota and human cells. By integrating traditional medicinal knowledge and modern pharmacological analysis, we undertook to support the potential use of BEO as a safe and effective prophylactic and therapeutic agent for vaginitis.

## 2. Materials and Methods

### 2.1. Plant Material Sourcing and Identification

Dried and rubbed basil (*Ocimum basilicum* L.) powder purchased from Shinyoung FS Co. (Gwangju, Republic of Korea) was freeze-dried using a lyophilizer (Rikakikai Co., Tokyo, Japan) under vacuum at −70 °C for 48 h to preserve heat-sensitive bioactive compounds.

### 2.2. Essential Oil Extraction

BEO was extracted by steam distillation, as previously described, with slight modifications [19]. Briefly, 400 g of dried basil powder was placed in a distillation apparatus (Dongguan City Niangge Machinery Co., Dongguan, China) containing 2 L of deionized water. Distillation was conducted at 100 ± 5 °C for 65 min or until no additional oil was collected. Modifications included adjusting the distillation time to optimize the oil yield and purity. The obtained BEO was dried over anhydrous sodium sulfate (Na_2_SO_4_) to remove residual moisture, and the extraction yield was calculated using the following equation:

Extraction yield (%) = [Weight of dried BEO obtained (g)/Weight of dried basil powder (g)] × 100.

### 2.3. Phytochemical Analysis

#### 2.3.1. Gas Chromatography–Mass Spectrometry (GC-MS)

The chemical composition of the BEO was analyzed by GC-MS (Agilent, Little Falls, DE, USA). GC was performed with a DB-5MS capillary column (30 m × 0.25 mm i.d. × 0.25 μm film thickness; 5%-phenyl-methylpolysiloxane stationary phase) using helium as the carrier gas, at a flow rate of 1 mL/min and an injector temperature of 220 °C.

The injection volume was 1 µL, and a split injection technique (split ratio 10:1) was used. The oven temperature was programmed as follows: 40 °C for 2 min, 40 °C to 240 °C at 15 °C/min, and 240 °C to 280 °C at 30 °C/min. MS was performed using an ion source temperature of 200 °C and a mass range of 40–400 *m*/*z*. The total run time was 18 min, and data acquisition was initiated 5 min after starting the runs. Compounds were identified by comparing the relative retention times and mass spectra with those in the Wiley and NIST (National Institute of Standards and Technology) libraries (Gaithersburg, MD, USA).

#### 2.3.2. Fractionation of BEO by Preparative HPLC

BEO was fractionated using a preparative HPLC system (LC-forte/R, YMC Co., Kyoto, Japan) equipped with a YMC-Triart Prep C18-S column (250 mm × 10.0 mm, i.d. 10 µm; YMC Co., Ltd., Kyoto, Japan). Prior to the separations, the column was equilibrated for 10 min at room temperature. The BEO was diluted to 0.3% using HPLC-grade methanol and filtered through a 0.45 µm membrane filter. A 100 µL aliquot of this solution was then injected into the system. The mobile phase consisted of solvent A (50% methanol in HPLC-grade water) and solvent B (HPLC-grade acetonitrile). The elution gradient was programmed using a linear gradient (solvent A 70% to 10% over 0–20 min), and the flow rate was maintained at 4.8 mL/min. Peak detection was performed using a photodiode-array detector (PDA) at 200 nm, 260 nm, and 282 nm to target different classes of bioactive compounds. Data acquisition and preprocessing were conducted using Clarity™ chromatography software (version 8.7.1.19, DataApex, Prague, Czech Republic).

### 2.4. Antioxidant Activity Assays

#### 2.4.1. DPPH Radical Scavenging Activity

DPPH (1,1-diphenyl-2-picrylhydrazyl) radical scavenging activity was assessed using a modified version of the Blois method [20]. Initially, a 60 µM DPPH solution was prepared in 95% ethanol, and the BEO sample was dissolved in dimethyl sulfoxide (DMSO; Duksan Pure Chemicals Co., Ltd., Ansan, Republic of Korea). The assay was conducted by mixing 100 µL of the DPPH solution with 100 µL of a serially diluted BEO solution in a 96-well microplate and incubating the mixture for 30 min in the dark at room temperature. Absorbance was measured at 540 nm using a microplate reader (TECAN Sunrise, Salzburg, Austria).

#### 2.4.2. ABTS Radical Scavenging Activity

The total antioxidant activity of basil essential oil (BEO) was measured using the ABTS (2,2′-azino-bis(3-ethylbenzothiazoline-6-sulfonic acid)) decolorization assay, as described by Re et al. [21], with slight modifications. Briefly, a 7 mM ABTS solution in distilled water was mixed with 2.45 mM potassium persulfate (K_2_S_2_O_8_) and incubated for 12 h in the dark at room temperature to generate ABTS radicals. The resulting ABTS radical cation solution was diluted with 5 mM phosphate-buffered saline (PBS, pH 7.4) to an absorbance of 0.70 ± 0.02 at 734 nm. For the assay, 990 µL of this diluted solution was mixed with 10 µL of the BEO sample solution in a 1.5 mL microcentrifuge tube and incubated for 6 min in the dark. The absorbance was measured at 734 nm using a spectrophotometer (TECAN Sunrise) (Männedorf, Switzerland).

### 2.5. Antibacterial Testing

#### 2.5.1. Strains and Culture Conditions

*G. vaginalis* (KCTC 5096), *F. vaginae* (KCTC 15240), and *C. albicans* (KCTC 7965) were obtained from the Korean Collection for Type Cultures (KCTC, Jeongeup, Republic of Korea). *C. gleum* (JCM 2410) was generously provided by Professor Geun Bae Kim of the Department of Microbiology, Chung-Ang University, Seoul, Republic of Korea. The microorganisms were cultured in an incubator (Jisico Co., Ltd., Seoul, Republic of Korea) at 37 °C under the following specific conditions:

*G. vaginalis* and *F. vaginae* were grown in tryptic soy broth (TSB; Difco Laboratories, Detroit, MI, USA) supplemented with 5% sheep blood (KisanBio, Seoul, Republic of Korea) and agar powder (Junsei Chemical Co., Tokyo, Japan). Cultures were incubated anaerobically for 24 h in an anaerobic chamber to maintain optimal growth conditions. *C. gleum* and *C. albicans* were cultured for 24 h in nutrient broth and YM broth, respectively, both solidified with agar (Difco Laboratories) (Detroit, MI, USA).

#### 2.5.2. Disk Diffusion Test

The antibacterial and antifungal activities of BEO against *G. vaginalis*, *F. vaginae*, *C. gleum*, and *C. albicans* were assessed using the paper disk–agar plate method, as described by De and Sherwood [22], with slight modifications. The microorganisms were sub-cultured three times and incubated in the selected liquid broth for 12 h. Aliquots (100 µL) of the broth, containing activated microorganisms adjusted to an optical density of 0.1 at 600 nm, were spread on corresponding agar plates. Sterile 8 mm paper disks (ADVANTEC, Tokyo, Japan) were inoculated with 50 µL BEO samples at concentrations of 0, 0.5, 1, 2, or 5 mg/disk. The diameters of the inhibition zones (mm) were measured in triplicate using a digital caliper (BD500-150, Bluetec, Seoul, Republic of Korea) after incubation for 12 to 24 h.

#### 2.5.3. Minimum Inhibitory Concentration (MIC)

MIC assays were conducted using the broth microdilution method, as described by Liu et al. [23]. Initially, microbial suspensions were standardized to an absorbance of 0.1 at 600 nm using a spectrophotometer (TECAN Sunrise). Each microorganism underwent three sequential sub-cultures and a 24 h activation period at 37 °C in the appropriate broth.

Serial twofold dilutions of the samples were prepared; methanol served as the negative control. In sterile 96-well plates (SPL Life Sciences, Pocheon, Republic of Korea), 198 µL bacterial suspensions were combined with 2 µL of serially diluted BEO solution. The MICs were defined as the lowest concentration that inhibited visible microbial growth, as indicated by the absence of turbidity. Microbial growth was also assessed by measuring absorbance at 600 nm to ensure accuracy, and all tests were performed in triplicate.

#### 2.5.4. Relative Microbial Growth Inhibition

Relative microbial growth inhibition was evaluated using a broth microdilution assay, as described by Liu et al. [23]. The microbial suspensions were adjusted to an absorbance of 0.1 at 600 nm and transferred to round-bottomed tubes. BEO samples were added at concentrations ranging from 62 to 500 µg/mL and incubated for 24 h at 37 °C in a shaking incubator (Vision Scientific, Daejeon, Republic of Korea). The microbial suspensions’ turbidities were measured using a spectrophotometer (TECAN Sunrise). All tests were performed in triplicate to ensure reproducibility. Relative microbial growth inhibition percentages were calculated using the following equation:

Relative microbial growth inhibition (%) = [1 − (Abs_sample_/Abs_control_)] × 100.

### 2.6. Toxicity Assessment

#### 2.6.1. Relative Microbial Viability of Beneficial Bacteria

The microbial viability assay was conducted using *L. crispatus* KCTC 5054 grown anaerobically for 24 h at 37 °C. Following activation, the liquid culture medium was continuously shaken for 12 h. BEO was added to MRS broth (Difco Laboratories) at concentrations ranging from 0 to 2000 µg/mL.

Microbial viability was assessed using the broth dilution method. Briefly, bacterial suspensions and BEO samples were added to the wells of a 96-well plate (SPL Life Sciences) and incubated for 24 h at 37 °C. The absorbances were measured at 600 nm in triplicate using a spectrophotometer (TECAN Sunrise). Relative microbial viability percentages were calculated using the following equation:

Relative microbial viability (%) = (Abs_sample_/Abs_control_) × 100.

#### 2.6.2. Cytotoxicity of BEO

Human dermal fibroblasts (HDFs) were established from the dermis of young foreskin or adult skin obtained at different anatomical locations. HDFs and human female reproductive tract epithelial cells (HeLa cells) were obtained from the American Type Culture Collection (ATCC, Manassas, VA, USA). The cells were cultured in Dulbecco’s Modified Eagle Medium (DMEM) supplemented with 10% fetal bovine serum (FBS), 2 mM glutamine, 100 U/mL penicillin, and 100 µg/mL streptomycin under a humidified 5% CO_2_ and 95% air atmosphere at 37 °C. Cell viability was assessed using the MTT assay.

### 2.7. Statistical Analysis

Statistical analyses were conducted using SPSS Statistics Version 26 (IBM Corp., Armonk, NY, USA). The results are presented as means ± standard deviations (SDs). One-way analysis of variance (ANOVA) followed by Duncan’s multiple range test was used to evaluate the effects of BEO treatment. Statistical significance was accepted for *p* values < 0.05.

## 3. Results

### 3.1. Composition of BEO by GC-MS

**Figure 1:**
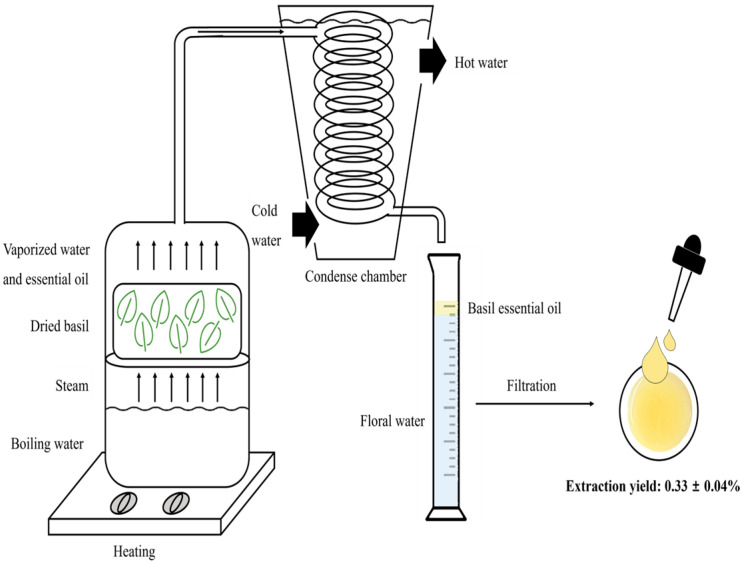
Schematic of the steam distillation process for basil essential oil extraction.

The BEO collected by steam distillation was a yellow essential oil with a characteristic aroma. The yield of BEO obtained was 0.33 ± 0.04% (*w*/*w*), and the oil was stored in vials at −20 °C in the dark (Figure 1).

The chemical composition of the BEO was determined by GC-MS. Twenty-one compounds were identified (Table 1). The primary constituents were estragole (29.48%), linalool (19.60%), methyl cinnamate (16.72%), trans-α-bergamotene (7.67%), and eucalyptol (6.33%). The BEO also contained various monoterpenes, phenylpropanoids, and sesquiterpenes, which are known to contribute to its antioxidant and antimicrobial properties [24,25,26].

### 3.2. Isolation and Identification of Antimicrobial Compounds

**Figure 2:**
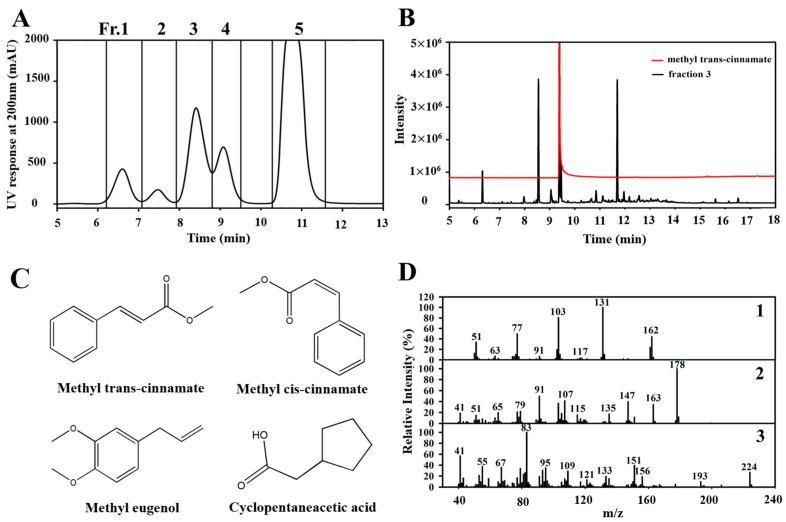
(**A**) Preparative HPLC chromatogram of basil essential oil. (**B**) GC-MS total ion chromatogram of Fraction 3 from basil essential oil and methyl trans-cinnamate. (**C**) Chemical structure of main constituents in Fraction 3. (**D**) Mass spectra of the three peaks of Fraction 3 (1 = methyl cis-cinnamate; 2 = methyleugenol; 3 = cyclopentaneacetic acid).

To isolate the antimicrobial compounds responsible for BEO’s activity, the oil was fractionated by preparative HPLC into five fractions (Fr. 1–5) (Figure 2A). The peaks were collected in separate tubes, and the solvent was removed by evaporation before further analysis.

**Figure 3:**
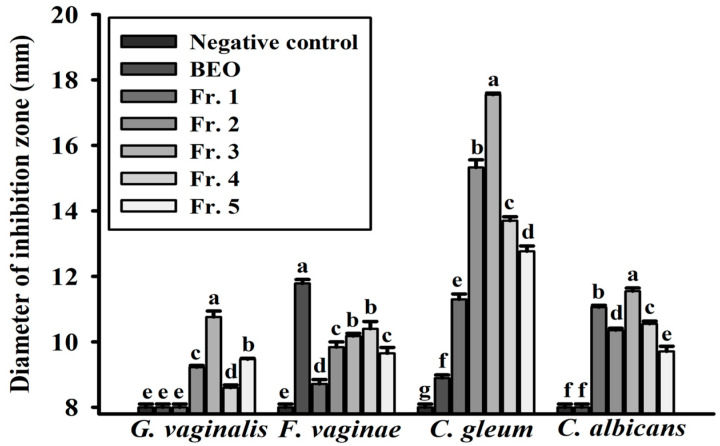
Disk diffusion test of basil essential oil and fractions: The pathogens investigated were *Gardnerella vaginalis* KCTC 5096, *Fannyhessea vaginae* KCTC 15240, *Chryseobacterium gleum* JCM 2410, and *Candida albicans* KCTC 7965. BEO: basil essential oil. Values are means ± standard deviations (SDs). ^a–g^ Values revealed significant differences using one-way analysis of variance followed by Duncan’s multiple range test (*p* < 0.05).

The antimicrobial activities of the BEO fractions were evaluated using paper disk diffusion tests, MIC assays, and a relative microbial growth inhibition assay. Fr. 3, which was rich in bioactive compounds, exhibited the greatest antimicrobial activity and produced large clear zones of inhibition against vaginitis-associated pathogens (Figure 3). The inhibition zones for Fr. 1–5 ranged from 8.60 ± 0.08 to 17.55 ± 0.05 mm at 0.5 mg/disk. The MIC results confirmed that Fr. 3 exhibited a significant inhibitory effect on all tested microorganisms, consistent with the disk diffusion assay findings (Table 2).

**Figure 4:**
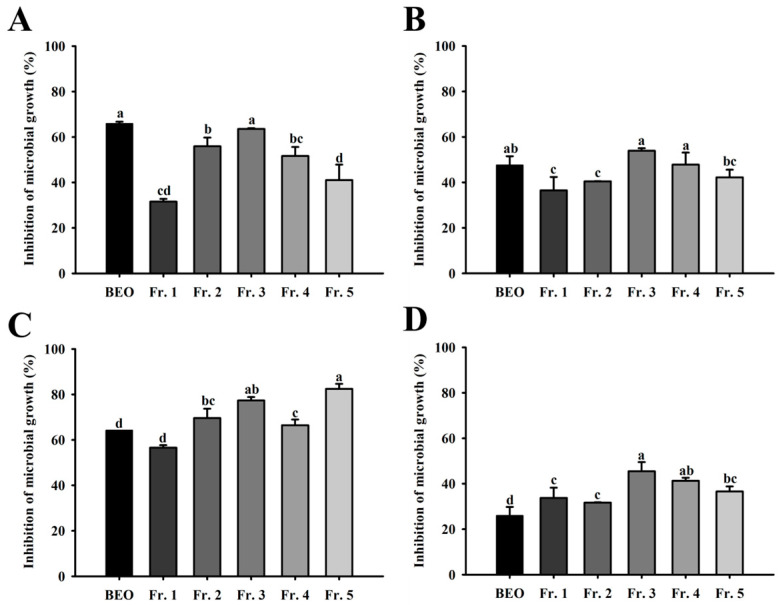
Relative microbial growth inhibition of basil essential oil and fractions: (**A**) Relative microbial growth inhibition against *Gardnerella vaginalis*. (**B**) Relative microbial growth inhibition against *Fannyhessea vaginae*. (**C**) Relative microbial growth inhibition against *Chryseobacterium gleum*. (**D**) Relative microbial growth inhibition against *Candida albicans*. Values are means ± standard deviations (SDs). ^a–d^ Values revealed significant differences using one-way analysis of variance followed by Duncan’s multiple range test (*p* < 0.05).

The relative microbial growth inhibition ratios of BEO and its fractions at 500 µg/mL against vaginitis pathogens, using methanol as a control, are shown in Figure 4. The results revealed that BEO and its five fractions exhibited relative microbial growth inhibition ratios ranging from 25.95% to 82.45%.

GC-MS (as described in Section 2.3.1) was used to analyze the primary antimicrobial and antifungal compounds responsible for the high activity of Fr. 3. The major peak in Fr. 3 showed a retention time and mass fragmentation pattern matching those of the methyl trans-cinnamate standard in the GC-MS analysis (Figure 2B). Figure 2C shows the chemical structures of the major compound in Fr. 3, methyl trans-cinnamate, along with other identified constituents including methyl cis-cinnamate, methyleugenol, and cyclopentaneacetic acid. Tandem mass fragmentation identified peaks at *m*/*z* 131 and 178, corresponding to methyl cis-cinnamate and methyleugenol, respectively (Figure 2D-1, 2), and cyclopentaneacetic acid was identified based on the presence of a fragment at *m*/*z* 83 (Figure 2D-3). The GC-MS profile of Fr. 3 showed a different composition compared to the BEO. This variation was expected, as preparative HPLC fractionation separates compounds based on their polarity and interaction with the stationary phase, resulting in the selective enrichment of specific constituents. In our case, methyl cinnamate, a relatively minor component in the crude oil, was highly concentrated in Fr. 3. Similar outcomes have been reported in studies of essential oil fractionation, where the chemical profiles of fractions differ significantly from that of the parent oil due to targeted isolation processes [27]. In the GC-MS analysis, eugenol was detected in the BEO, whereas methyl eugenol was identified as a major compound in Fr. 3. It is important to note that eugenol and methyl eugenol share a similar chemical structure and may elicit similar fragmentation patterns in GC-MS analysis. Although eugenol was detected in the crude oil, methyl eugenol may have been selectively enriched in Fr. 3 during the fractionation process due to differences in their polarity. Thus, the presence of methyl eugenol in Fr. 3 was likely a result of selective isolation during preparative HPLC. Likewise, cyclopentaneacetic acid, undetected in the crude oil, was found in significant amounts in Fr. 3. It is plausible that this compound, initially present at trace levels, was concentrated through HPLC fractionation. Additionally, its absence in the BEO chromatogram may be attributed to matrix complexity or detection limits of the GC-MS method.

### 3.3. Antioxidant Activities of BEO

Table 3 presents the EC_50_ values (half-maximal effective concentration) of BEO for DPPH and ABTS radical scavenging activities. The EC_50_ of DPPH was 115.36 ± 2.19 µg/mL, while the ABTS assay produced different results.

### 3.4. Disk Diffusion Assays of BEO Against Vaginitis Pathogens

A disk diffusion assay was used to evaluate the antimicrobial activities of BEO against vaginitis-associated pathogens, and the results showed that the BEO exhibited strong dose-dependent antimicrobial effects against all four tested pathogens (Table 4). The inhibition zones ranged from 13.75 ± 0.39 mm to 25.88 ± 0.52 mm at the highest concentration tested (5 mg/disk). In addition, the BEO displayed broad-spectrum effects and inhibited Gram-positive (*G. vaginalis* and *F. vaginae*) and Gram-negative bacteria (*C. gleum*), as well as *C. albicans* (a dimorphic fungus).

### 3.5. Evaluation of the MIC Values of BEO

The MIC values of BEO were determined using a broth microdilution assay to assess its antimicrobial activities against the four pathogens. The MIC was defined as the lowest concentration of BEO that inhibited visible bacterial growth after 24 h of incubation at 37 °C. The BEO effectively inhibited all tested pathogens, with MIC values ranging from 31 to 500 µg/mL (Table 4).

### 3.6. Relative Microbial Growth Inhibition of Vaginitis Pathogens by BEO

**Figure 5:**
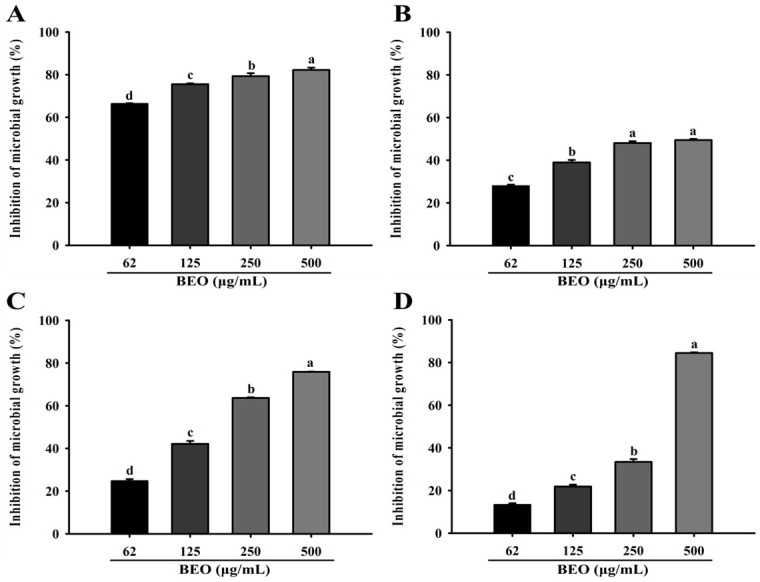
Relative microbial growth inhibition of basil essential oil against vaginitis pathogens: (**A**) Relative microbial growth inhibition toward *Gardnerella vaginalis*. (**B**) Relative microbial growth inhibition toward *Fannyhessea vaginae*. (**C**) Relative microbial growth inhibition toward *Chryseobacterium gleum*. (**D**) Relative microbial growth inhibition toward *Candida albicans*. Values are means ± standard deviations (SDs). ^a–d^ Values revealed significant differences using one-way analysis of variance followed by Duncan’s multiple range test (*p* < 0.05).

Microbial growth inhibition by BEO was assessed spectrophotometrically, and the results obtained showed it had dose-dependent effects against *G. vaginalis*, *F. vaginae*, *C. gleum*, and *C. albicans*. At concentrations of 62, 125, 250, and 500 µg/mL, the relative microbial inhibition ratios against *G. vaginalis* were 66.44 ± 0.23%, 75.55 ± 0.33%, 79.31 ± 1.39%, and 82.21 ± 1.13%, respectively. Against *F. vaginae* and *C. gleum*, the inhibition ratios ranged from 28.02 ± 0.55% to 75.86 ± 0.12%. At 500 µg/mL, the BEO inhibited the growth of *C. albicans* by 84.46 ± 0.32% (Figure 5).

### 3.7. Safety Assessment of BEO

**Figure 6:**
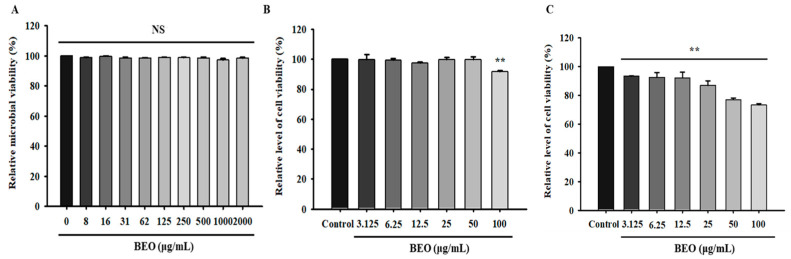
(**A**) Relative microbial viability of *Lactobacillus crispatus* after exposure to basil essential oil. (**B**) The cell viability of HDFs after exposure to basil essential oil. (**C**) The cell viability of HeLa cells after exposure to basil essential oil. NS: not significant; ** *p* < 0.05 vs. untreated controls. Values are means ± standard deviations (SDs).

Ensuring the safety of BEO for topic applications, such as skin cleansers, is essential. In this study, we evaluated its toxic effects on *L. crispatus* and human dermal cells. To quantitatively assess microbial viability, relative microbial viability was determined spectrophotometrically after 24 h of BEO exposure. The BEO did not significantly affect the viability of *L. crispatus*, and viability remained above 95% at all tested concentrations (Figure 6A).

To further assess the safety of BEO for skin applications, we examined its effects on human dermal fibroblasts (HDFs) and HeLa cells [28]. HDFs and HeLa cells were incubated with BEO at concentrations ranging from 3.125 to 100 µg/mL for 24 h, and cell viabilities were determined using the MTT assay. No significant reduction in cell viability versus untreated controls was observed at concentrations of 3.125–50 µg/mL. Furthermore, when HDFs were exposed to BEO at 100 µg/mL, cell viability remained high at 91.88 ± 0.67% (Figure 6B), and for HeLa cells, viability ranged from 93.33 ± 0.30% to 73.23 ± 1.11% across the same concentration range, indicating a dose-dependent decrease at higher concentrations (Figure 6C).

## 4. Discussion

The findings of this study are consistent with those of previous studies, although source-dependent variations in the chemical composition of basil essential oil (BEO) have been reported. Chalchat and Özcan reported high concentrations of estragole (52.60%) and limonene (13.64%) in BEO [29], whereas Kavoosi and Amirghofran identified linalool (31.65%), estragole (17.37%), and methyl cinnamate (15.14%) as the major constituents [30].

The chemical composition of BEO, which includes methyl trans-cinnamate, methyl cis-cinnamate, methyleugenol, and cyclopentaneacetic acid, is probably responsible for its observed antimicrobial and antioxidant activities. Methyl trans-cinnamate, the simplest-structured cinnamate ester, is a significant component in basil and other *Ocimum* species (Figure 2C) [31,32]. This phytochemical is known for its anti-inflammatory, anti-obesity, anticancer, and antimicrobial properties and is widely utilized in the commodities industry [33,34,35,36]. Furthermore, the identification of these bioactive compounds in BEO suggests that multiple constituents may contribute to the antimicrobial effect of BEO. Synergistic effects, if any, require further investigation through combination testing of individual components. Discrepancies in the chemical composition and activity of BEO may be caused by differences in agroclimatic factors such as climate, season, and geographical conditions, which all influence basil cultivation [37].

Similarly, previous studies have shown that phytocompounds have meaningful antimicrobial activities. Jang et al. reported that the antibacterial activity of the neutral fraction of rhubarb (*Rheum rhabarbarum*) against *C. gleum* resulted in an inhibitory zone of 10 mm at a concentration of 10 mg/mL [38]. Similarly, Marcas et al. demonstrated that 20 µL of *Bixa orellana* L. leaf extract produced inhibition zones of 20.3 mm and 12.0 mm against *G. vaginalis* and *F. vaginae*, respectively [39]. Furthermore, Ardestani et al. reported that ajwain (*Trachyspermum ammi*) essential oil exhibited significant antimicrobial activity against various vaginitis pathogens, with MIC values ranging from 31.5 to 500 µg/mL [40].

While the specific composition and concentrations of active compounds vary across plant species, these findings, along with our own results, support the broader trend that essential oils from diverse botanical sources can exert significant inhibitory effects against vaginal pathogens. The potency of BEO, particularly its low MIC value and broad-spectrum activity, highlights its potential utility as a superior natural antimicrobial agent.

Although Fr. 3 exhibited potent antimicrobial activity in the MIC and microbial growth inhibition assays, the BEO showed stronger inhibition against *F. vaginae* in the disk diffusion assay. This discrepancy may be attributed to synergistic or additive effects among the multiple constituents in the whole oil, which may be diminished upon fractionation. It is also worth noting that the disk diffusion method has inherent limitations, particularly in evaluating the activity of less volatile or poorly diffusing compounds, and therefore may underestimate the potency of isolated fractions.

Previous reports have emphasized the critical role of phenolic compounds—such as thymol and eugenol—in antimicrobial activity, especially against vaginal pathogens [41,42,43]. Phenolic compounds are also known for their antioxidant effects in cosmetology, largely due to the number and position of hydroxyl groups in their chemical structures [44]. Incorporating such antioxidants in cosmetic formulations can prevent oxidation of lipid components and vitamins [45], suggesting that phytocompounds like eugenol may contribute to both antimicrobial and antioxidant efficacy. In our study, the absence or low abundance of phenolic constituents in Fr. 3 may partially explain its comparatively lower inhibition zone.

In addition, it is essential to ensure the safety of products like BEO intended for human applications. Our cytotoxicity assessments on HDFs and HeLa cells confirmed the biocompatibility of BEO at concentrations that exhibited potent antimicrobial activity. These findings align with those of Syarina et al., who reported that *Spirulina platensis* extract, when applied to HDFs at 50 µg/mL for 24 h, maintained cell viability greater than 80% [46]. Thus, our findings demonstrate that BEO has potential use for skin application when used at appropriate dosages.

## 5. Conclusions

BEO demonstrated potent antimicrobial activity against four vaginitis-associated pathogens, viz., *G. vaginalis*, *F. vaginae*, *C. gleum*, and *C. albicans*, and it had minimal cytotoxic effects on *L. crispatus* and human dermal fibroblasts. Methyl trans-cinnamate was identified as the primary active compound in BEO, which supports the rationale underlying the traditional medicinal use of *Ocimum* species. These findings suggest that BEO has significant potential as a natural therapeutic agent for vaginitis and should be subjected to further in vivo and clinical investigations.

## Figures and Tables

**Table 1:** Chemical composition of the basil essential oil as determined by GC-MS; n.i: non-identified.

No.	Compound	Formula	Rt (min)	Area (%)
1	Bicyclo[3.1.1]heptane	C_7_H_12_	5.080	0.67
2	2-Isopropyltoluene	C_10_H_14_	5.586	0.48
3	l-Limonene	C_10_H_16_	5.647	0.45
4	Eucalyptol	C_10_H_18_O	5.714	6.33
5	Linalool	C_10_H_18_O	6.440	19.60
6	Camphor	C_10_H_16_O	7.059	0.37
7	Estragole	C_10_H_12_O	7.593	29.48
8	(-)-Carvone	C_10_H_14_O	8.057	0.38
9	Bornyl acetate	C_12_H_20_O_2_	8.479	1.21
10	Methyl cinnamate	C_10_H_10_O_2_	8.642	2.37
11	Eugenol	C_10_H_12_O_2_	9.141	0.44
12	Methyl cinnamate	C_10_H_10_O_2_	9.519	16.72
13	Bicyclo[5.2.0]nonane	C_15_H_24_	9.549	1.72
14	Caryophyllene	C_15_H_24_	9.933	1.07
15	trans-alpha-Bergamotene	C_15_H_24_	9.973	7.67
16	alpha-Humulene	C_15_H_24_	10.269	0.53
17	n.i	-	10.470	0.48
18	Germacrene-D	C_15_H_24_	10.493	1.88
19	n.i	-	10.627	0.42
20	n.i	-	10.650	0.46
21	gamma-Amorphene	C_15_H_24_	10.759	2.59
22	(E)-alpha-Bisabolene	C_15_H_24_	10.904	0.80
23	(-)-Caryophyllene oxide	C_15_H_24_O	11.436	0.30
24	n.i	-	11.673	0.64
25	Epi-cadinol	C_15_H_26_O	11.883	2.94
				100.00

**Table 2:** The MIC values of basil essential oil fractions against vaginitis pathogens. The pathogens investigated were *Gardnerella vaginalis* KCTC 5096, *Fannyhessea vaginae* KCTC 15240, *Chryseobacterium gleum* JCM 2410, and *Candida albicans* KCTC 7965. MIC: minimum inhibitory concentration. BEO: basil essential oil.

	MIC	BEO	Fr. 1	Fr. 2	Fr. 3	Fr. 4	Fr. 5
*G. vaginalis*	(μg/mL)	125	125	62	31	62	125
*F. vaginae*	(μg/mL)	31	250	250	31	125	125
*C. gleum*	(μg/mL)	31	125	62	16	62	16
*C. albicans*	(μg/mL)	500	250	250	62	125	125

**Table 3:** Antioxidant activities of basil essential oil. DPPH: 1,1-diphenyl-2-picrylhydrazyl. ABTS: 2,2-azono-bis-3-ethylbenzthiazoline-6-sulphonate. EC_50_: The half-maximal effective concentration. Values are means ± standard deviations (SDs).

DPPH	ABTS
EC_50_ (μg/mL)	
115.36 ± 2.19	54.77 ± 0.29

**Table 4:** Disk diffusion test for antimicrobial activity against vaginitis pathogens: The pathogens investigated were *Gardnerella vaginalis* KCTC 5096, *Fannyhessea vaginae* KCTC 15240, *Chryseobacterium gleum* JCM 2410, and *Candida albicans* KCTC 7965. Values are means ± standard deviations (SDs). ^a–d^ Values significantly different according to one-way analysis of variance followed by Duncan’s multiple range test (*p* < 0.05). MIC: minimum inhibitory concentration.

	Diameter of Inhibition Zone (mm)					MIC
Test Strains	mg/disk					μg/mL
	0	0.5	1	2	5	
*G. vaginalis*	8.02 ± 0.01 ^a^	8.03 ± 0.02 ^c^	9.91 ± 0.35 ^c^	11.79 ± 0.20 ^b^	13.75 ± 0.39 ^d^	125
*F. vaginae*	8.03 ± 0.01 ^a^	12.97 ± 0.11 ^a^	19.34 ± 0.46 ^a^	22.71 ± 0.33 ^a^	25.88 ± 0.52 ^a^	31
*C. gleum*	8.03 ± 0.01 ^a^	8.09 ± 0.01 ^b^	11.48 ± 0.13 ^b^	13.06 ± 0.02 ^b^	17.74 ± 0.25 ^b^	31
*C. albicans*	8.02 ± 0.01 ^a^	8.02 ± 0.02 ^c^	9.03 ± 0.10 ^d^	11.07 ± 0.30 ^d^	13.95 ± 0.11 ^c^	500

## Data Availability

Data are contained within the article.

## Abbreviations

ABTS	2,2′-Azino-bis(3-ethylbenzothiazoline-6-sulfonic acid)
ANOVA	Analysis of variance
ATCC	American Type Culture Collection
BEO	Basil essential oil
BV	Bacterial vaginosis
DMEM	Dulbecco’s Modified Eagle Medium
DMSO	Dimethyl sulfoxide
DPPH	1,1-Diphenyl-2-picrylhydrazyl
EC_50_	Half-maximal effective concentration
FBS	Fetal bovine serum
GC-MS	Gas chromatography–mass spectrometry
HDF	Human dermal fibroblast
NIST	National Institute of Standards and Technology
MIC	Minimum inhibitory concentration
PBS	Phosphate-buffered saline
PDA	Photodiode-array detector
SD	Standard deviation
TSB	Tryptic soy broth
VVC	Vulvovaginal candidiasis
